# On the Realistic Radio and Network Planning of IoT Sensor Networks

**DOI:** 10.3390/s19153264

**Published:** 2019-07-24

**Authors:** Loizos Kanaris, Charalampos Sergiou, Akis Kokkinis, Aris Pafitis, Nikos Antoniou, Stavros Stavrou

**Affiliations:** 1 Department of Electrical Engineering, Eindhoven University of Technology, 5600 Eindhoven, The Netherlands; 2 Department of Computer Science, University of Cyprus, 2109 Nicosia, Cyprus; 3 Department of Electrical Engineering, Computer Engineering and Informatics, Cyprus University of Technology, 3036 Limassol, Cyprus; 4 Research and Development Department, Sigint Solutions Ltd., 2311 Nicosia, Cyprus; 5 Faculty of Pure and Applied Sciences, Open University of Cyprus, 2220 Nicosia, Cyprus

**Keywords:** WSN, IoT, radio planning, physical layer, network layer, simulation, sensor, Cooja, TruNET wireless

## Abstract

Planning and deploying a functional large scale Wireless Sensor Network (WSN) or a Network of Internet of Things (IoTs) is a challenging task, especially in complex urban environments. A main network design bottleneck is the existence and/or correct usage of appropriate cross layer simulators that can generate realistic results for the scenario of interest. Existing network simulators tend to overlook the complexity of the physical radio propagation layer and consequently do not realistically simulate the main radio propagation conditions that take place in urban or suburban environments, thus passing inaccurate results between Open Systems Interconnection (OSI) layers. This work demonstrates through simulations and measurements that, by correctly passing physical information to higher layers, the overall simulation process produces more accurate results at the network layer. It is demonstrated that the resulting simulation methodology can be utilized to accomplish realistic wireless planning and performance analysis of the deployed nodes, with results that are very close to those of real test-beds, or actual WSN deployments.

## 1. Introduction

The main radio propagation mechanisms taking place in outdoor and/or indoor suburban or urban areas include reflections, transmissions, diffraction and in certain occasions scattering. Empirical or semi-empirical radio propagation modelling techniques can be limited in nature, can be used only in similar environments and conditions like the ones used to develop the models and tend to suffer from high standard deviations around the mean received signal strength (RSS) value. These type of models do have their usefulness when one is not interested in highly accurate predictions but instead is interested in fast predictions. Still, when the wireless system performance is characterised by a bimodal behaviour, i.e., in wireless sensor or IoT networks based on Zigbee or 802.15.4 technologies, accurate prediction results at the edge of coverage are of paramount importance since miscalculations can lead to non-functional Wireless Sensor Networks (WSNs) or IoT Network deployments. Instead, in order to avoid such pitfalls, the wireless network designer can utilise deterministic radio planning tools that can increase simulation accuracy [1,2], since significant differences exist between simplified theoretical performance and actual deployments [3]. This fact imposes significant challenges in designing and developing smart cities, where the efficient operation of large scale, heterogeneous Wireless Sensor Networks is mandatory [4].

Existing wireless network design tools/simulators may fail in providing functional deployed networks due to their inability to simulate realistically at the physical layer the performance of complex wireless network setups, i.e., their RSS coverage or any interference conditions. Existing wireless network layer simulators tend to implement and use simplistic propagation models, such as free space loss, plane earth loss, or utilize statistical or semi-empirical models, which are inadequate to extract realistic coverage results [3]. Such simplified approaches lead to miscalculation of radio coverage parameters and erroneous predictions of radio propagation characteristics which subsequently influence prediction performance at the network layer, resulting in erroneous network performance predictions.

In this work, we present the result analysis of the proposed simulation methodology that combines results from two simulators that operate at different OSI layers for the purpose of increasing simulation accuracy. The first simulator, TruNET wireless [5], is a realistic 3D polarimetric physical layer simulator capable of accurately simulating radio propagation effects in urban and suburban scenarios based on advanced ray tracing techniques. The second simulator, Cooja [6], is an open source network simulator for the Contiki open source operating system especially designed for WSN or Internet of Things networks. With Cooja, large and small networks of Contiki nodes can be simulated. Nodes can be also emulated at the hardware level, which is slower, but allows precise inspection of the system behavior, or at a less detailed level, which is faster and allows simulation of larger networks. Finally, Cooja has the unique advantage that the simulated source code can be downloaded and run into real nodes. By combining the two simulators, realistic physical predictions from realistic simulated environments in TruNET can be passed to Cooja, hence achieving accurate network performance estimations, which is critical for developing reliable WSN or IoT networks.

The proposed simulation methodology aims to create a realistic and functional cross layer wireless sensor network design platform, focusing on the deployment of WSNs and IoT networks, in smart city environments. The interconnection of the two aforementioned simulators offers the following advantages:

(a) A simplified procedure for generating a real city environment, based on either importing the 3D building data, or by creating the environment in the TruNET environment,
(b) configuring the constitutive, or electrical, parameters of various materials such as walls, glass, earth, metal, etc.,
(c) parameterization and deployment of a large number of sensors or interfering devices in the city environment, based on a set of dynamic rules,
(d) large scale physical layer simulation capability, using deterministic radio propagation models, ensuring realistic Received Signal Strength (RSS) coverage estimation, and interference prediction,
(e) accurate network layer performance calculation through tight integration with the Cooja network simulator.

## 2. Related Work

The design of complex IoT setups requires the support of large scale test-beds or the usage of scalable simulation tools [7].

A number of such tools and experimental frameworks, as proposed by the research community, is presented in literature. In [8], the authors present a framework that supports the parallel and distributed simulation of large scale, complex wireless system models. Results show that the proposed simulation framework is able to transparently adapt to the execution system and model characteristics, by dynamically reducing communication overhead, thus reducing simulation time and increasing simulation scalability. In [9], authors suggest a model-driven simulation (based on the standard language SDL) to describe an IoT scenario. Starting from this, an automatic code generation transforms the description into an executable simulation model for the ns-3 network simulator. In [10], researchers presented DPWSim, a simulation toolkit designed to support the development of service-oriented and event-driven IoT applications on top of IoT devices with secure Web service capabilities and a seamless integration into existing World Wide Web infrastructure applications. DPWSim allows developers to prototype, develop, and test IoT applications using the DPWS (Devices Profile for Web Services) technology without the presence of physical devices.

A notable work that reinforces the finding of our work is presented in [11] where authors measure the Received Signal Strength Indication (RSSI) of near-ground WSNs at 470 MHz under four different terrains (flat concrete road, flat grass, undulating grass and flat grass with obstacles) and obtained the corresponding path loss models. Through comprehensive analysis of the influence of different antenna heights and terrain factors, they showed the limit of existing theoretical models. Furthermore, they proposed a propagation model selection strategy to more accurately reflect the true characteristics of the near-ground wireless channels for WSNs. These models were implemented on Cooja simulator and showed that simplistic theoretical models would induce great inaccuracy of network connectivity estimation.

In [12], the authors research the functionality of compression and routing protocols in an IoT environment. In particular, they study how the COOJA network simulator enables the emulation of different kinds of nodes and how the routing matrices are computed. COOJA simulator is used as a power visualizer.

Finally, the authors of [13] present Cup Carbon, a multi-agent and discrete-event, WSN simulator. In this tool, networks can be designed in an ergonomic user-friendly interface using the Open Street Map (OSM) framework, where sensors are deployed directly on map and one can study the behaviour of a WSN network and its related cost. A more broad survey is presented in [3], where authors conclude that, despite the significant technological advances presented, the difficulties associated with the evaluation of IoT solutions under realistic conditions in real world experimental deployments, still hampers their maturation and significant roll out. Furthermore, in their work, they identify the requirements for the next generation of experimental IoT facilities. In particular, they compare 23 existing testbeds—simulations publicly accessible and with notable use, concluding that a new generation of IoT experimentation facilities is required.

In the aforementioned research work, a common drawback in proposed simulation tools/frameworks is the utilization of simplified, inaccurate propagation models for the estimation of physical layer parameters. Ignoring or miscalculating the behaviour of the physical layer renders the realistic planning and placement of IoTs in suburban or urban environments infeasible.

Similar issues also occur when utilizing built-in/default radio propagation models in typical WSNs simulators such as ns-2 [14], ns-3 [15], TOSSIM [16], OMNET++ [17], Cooja [6] and OPNET [18].

## 3. Objective

The main objective of this work is to examine and compare the simulation results obtained during the design of WSN and/or IoT networks when a deterministic 3D polarimetric radio planning simulator is interconnected with a typical network layer simulator. As we have already discussed in the previous section, currently, the capabilities of existing network simulators on realistically implementing the whole OSI network stack are limited. The majority of Network simulators focus on simulating algorithms and protocols starting from the MAC layer, or even from the network layer and above, while they tend to oversimplify the physical layer. Thus, such simulators will not necessarily produce results which are comparable to measurements from real wireless network deployments.

## 4. Proposed Approach

### 4.1. High Level Concept

**Figure 1:**
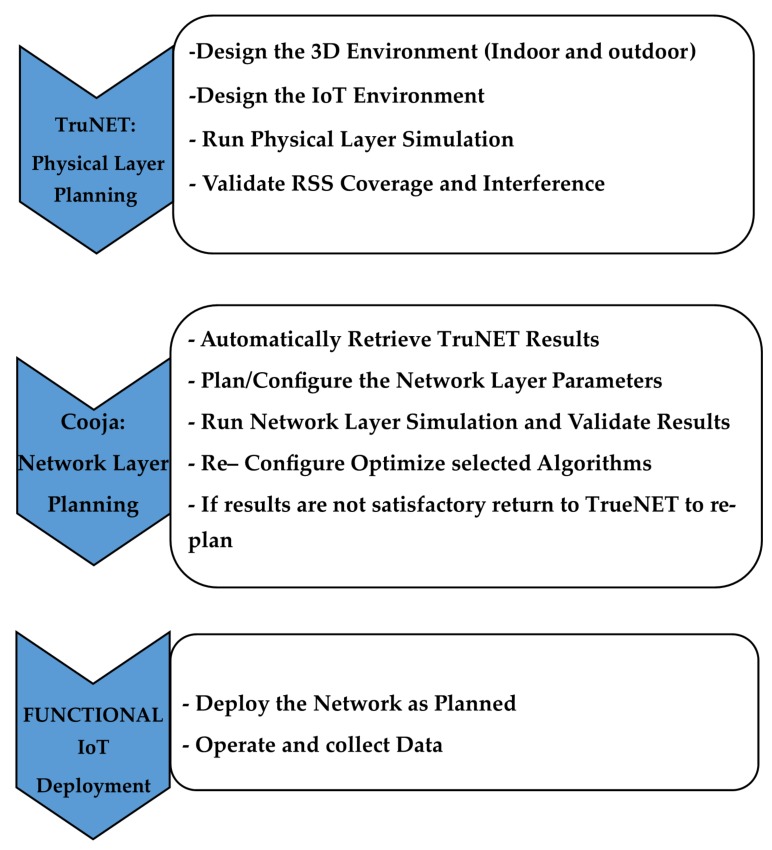
TruNET-Cooja interconnection: proposed methodology flowchart.

The proposed methodology creates a realistic snapshot of the Received Signal Strength (RSS) area coverage through TruNET which is then injected into Cooja, in order to generate a functional binary file that can be downloaded in real nodes for immediate implementation. In Figure 1, we present the flowchart of the proposed process for IoT sensor network deployment.

**Figure 2:**
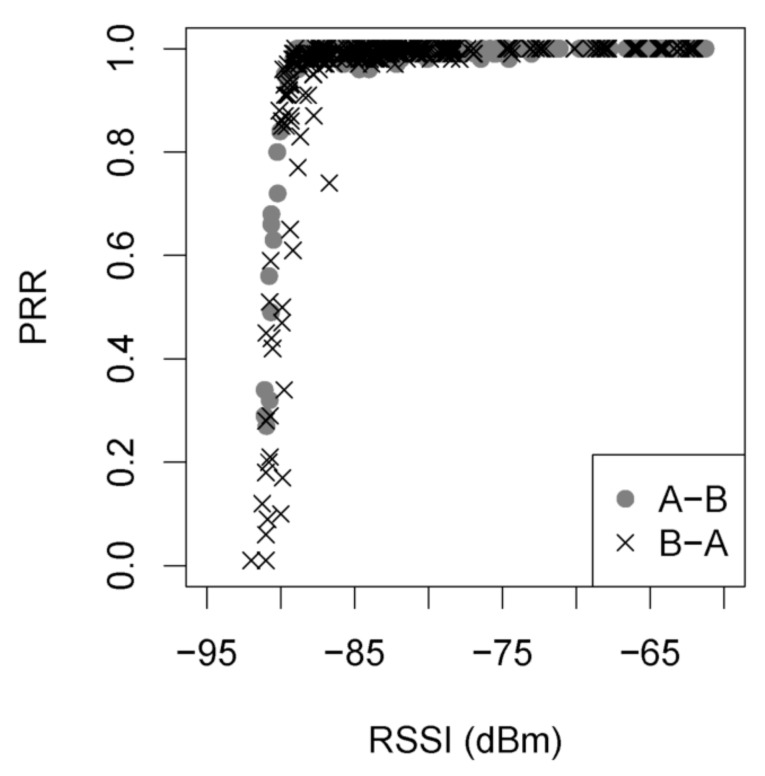
Packets reception rate vs. RSS [19].

The proposed process allows the 3D adjustment, visualisation and realistic simulation of wireless node deployment. The importance of accurately predicting the RSS coverage at the physical layer and combining it with the network layer is highlighted by the results of Figure 2.

In this figure, the Packet Reception Rate (PRR) vs. the RSS is presented, based on a measurement campaign performed in [19], for the same radio chip CC2420 that was used in this demo. It is clear that correlation between PRR and RSS varies with an obvious transition. When the RSS is better than -87 dBm, the PRR is always beyond 90%, indicating a desirable link; when the RSS is less than -92 dBm, the PRR is close to zero. In the transition zone, packets may also be lost due to instantaneous interference conditions from other co-channel systems or nodes.

The aforementioned bimotal behaviourof Wireless Sensor Nodes highlights that, if the RSS coverage estimation is not accurate enough, the simulation will most probably indicate a misleading RSS coverage and thus network performance, while, when deployed in a real environment, the network may under-perform or completely fail if radio propagation characteristics are not accurately predicted.

### 4.2. Methodology Implementation

The proposed methodology was realized by implementing a dedicated export functionality into TruNET, which was dubbed as *Cooja DGRM Links*.

For Cooja-Contiki version 2.7, to work with the newly added functionality *Cooja DGRM Links*, a Java plugin file, named *DGRMConfigurator27TruNET.java*, was also created. This file was then copied in the respective plugin folder and registered in the plugins line.

From this point forward, Cooja is able now to support, i.e., import, the exported realistic physical layer data from TruNET wireless. When Cooja launches, a new simulation can be created utilizing the “Directed Graph Radio Medium (DGRM)” as the selected radio model. Nodes are created as per user requirements and, at this step, the *Cooja DGRM Links (for TruNET wireless)* should be selected, in order to import the corresponding TruNET file.

## 5. Experimental Setup and Test Environment

In order to evaluate the efficiency and reliability of the proposed methodology, the following experiment was performed.

### 5.1. Step I: Real IoT Deployment

**Figure 3:**
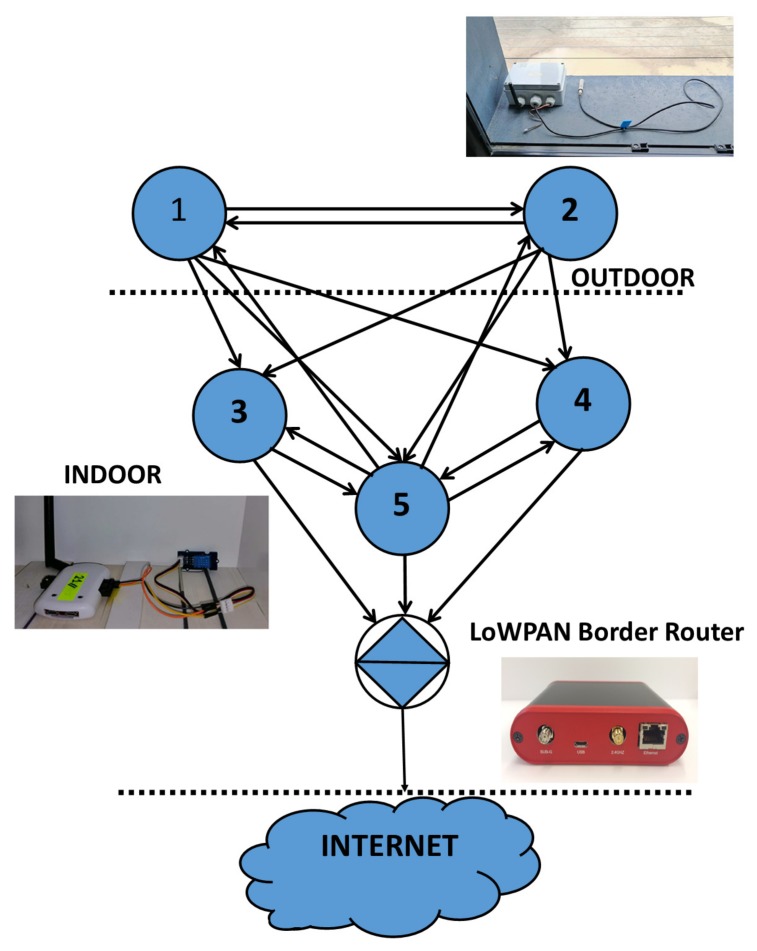
Network setup topology.

We deployed a real IoT network with Zolertia RE-Mote Revision B nodes. In the experimental setup, five nodes were placed in indoor and outdoor locations within and around a laboratory area of 100 m ${}^{2}$. The nodes were randomly relocated within the same area in order to increase the received sample size and ensure objective results [20]. In every topology setup, the nodes were connected in a LoWPAN Border Router and, from that point, to the Internet. A typical wireless topology utilized in the experiment is presented in Figure 3. RSS values were recorded for a period of one week, at 15-min intervals.

### 5.2. Step II: Physical Layer Simulations

The exact 3D building structure of the test environment, including the furniture, was designed in TruNET. All objects were configured and presented in Table 1. A detailed analysis of the Ray Tracing calibration procedure can be found in [21]. The wireless network topology, with the exact note parameters, was also designed.

All the scenarios were run, using the 3D polarimetric ray tracing model of TruNET wireless. This model is a 3D deterministic implementation of a Ray Launching Algorithm. Transmitting sensors launch a number of rays in the 3D domain. Every launched ray follows a distinct path between a transmitter and a receiver and interacts with the geometry of the defined environment. The electric field is calculated using the three-dimensional, electromagnetic formulation for Reflection, Refraction and Uniform Theory of Diffraction (UTD) [5].

**Figure 4:**
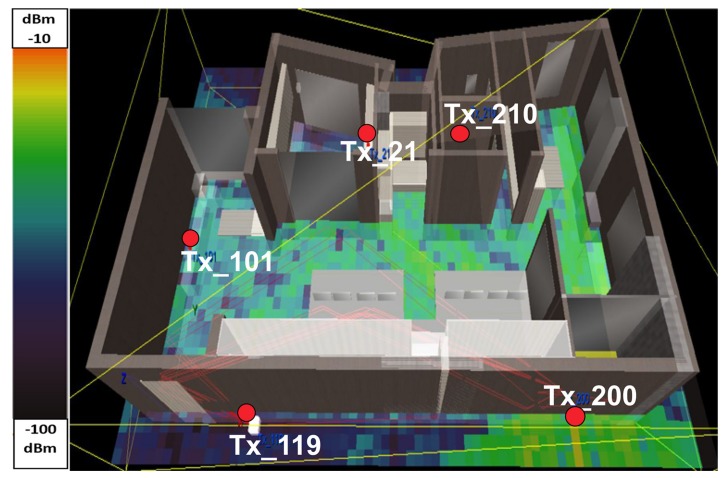
TruNET simulation of WSN coverage.

For every scenario, the RSS coverage map was recorded. A snapshot of the extracted radiomaps is depicted in Figure 4. The purpose of this step of the experiment is twofold: (a) to verify that the RSS values estimated by TruNET simulation are close to the actual measured RSS values and (b) to export the data in order to inject it into the Cooja simulator as per step III below.

### 5.3. Step III: Pure Network Layer and Interconnected Physical-Network Layer Simulations

During the third step of our experiment, we deployed the nodes in the Cooja simulator at the same locations, as the real nodes and the nodes deployed in the real TruNET platform, in terms of x,y,z location parameters. Initially, all the scenarios are run by selecting the built-in models *Unit Disk Graph Model (UDGM)* with the default settings and *Multi-path Ray-tracer Medium (MRM)*. The MRM configuration settings are presented in Table 2.

**Figure 5:**
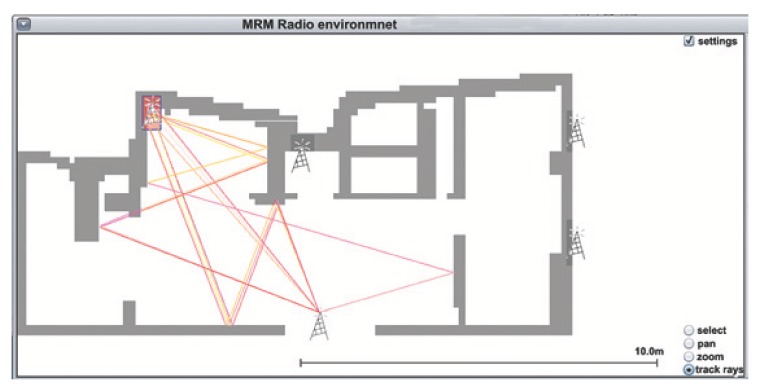
MRM model simulation.

A snapshot of the MRM model simulation is depicted in Figure 5.

**Figure 6:**
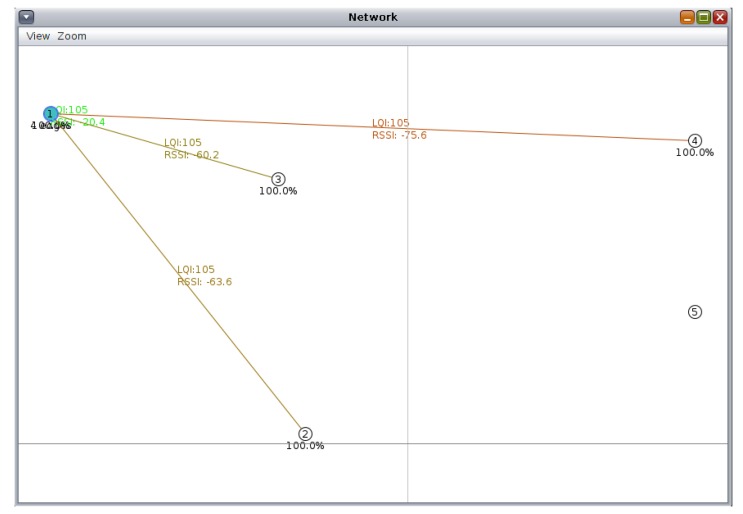
Cooja-TruNET interconnected simulation: WSN links.

Finally, the RSS coverage maps exported from TruNET (step 1) are injected into Cooja, as per the procedure described in Section 4.2, and the same wireless network topologies are re-run. A sample of the WSN links with the utilization of TruNET output results is shown in Figure 6.

## 6. Result Analysis

### 6.1. RSS Coverage

**Figure 7:**
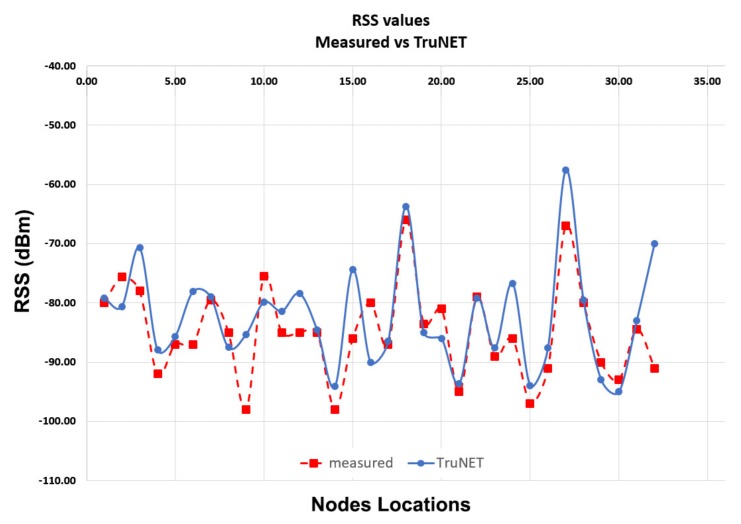
RSS: Measured vs. TruNET Simulation.

**Figure 8:**
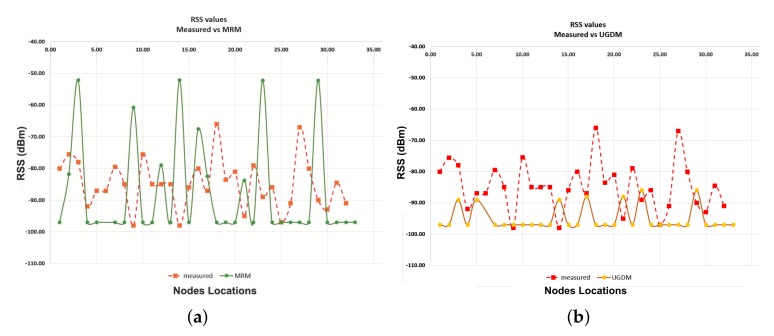
RSS: Measured vs. Cooja built-in Models Simulation (**a**) Measured vs. MRM Simulation; (**b**) Measured vs. UDMG Simulation.

This section analyses the RSS Coverage results of each sensor deployed in the area of interest. If RSS is accurately estimated, it will realistically affect the outcome of the network layer performance parameters, such as the packet delivery. A comparison between the measured RSS values and the simulation results produced from the three different simulation models is presented below. The comparison is depicted in Table 3 and Table 4 and graphically presented in Figure 7 and Figure 8. It can be clearly seen that the simulated RSS values from TruNET wireless highly approximate the measured ones, reaching a correlation level of 73%. On the contrary, both Cooja built-in models deviate significantly from the measured values (correlation level 25% and 26% respectively), hence failing to realistically predict whether a reliable communication link can be established between sensors.

**Figure 9:**
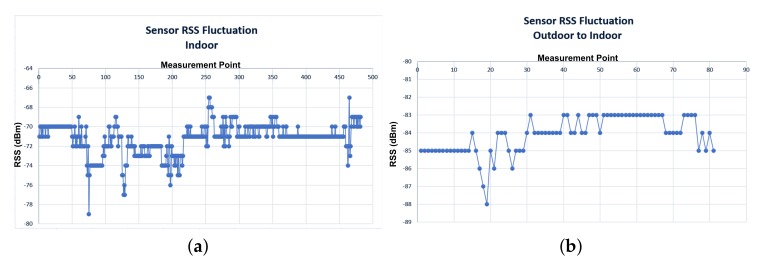
Measured RSS fluctuation of sensors (**a**) Indoor; (**b**) Outdoor.

In order to further analyse the typical RSS fluctuation behaviour in a deployed WSN, the RSS values of wireless nodes for an indoor and outdoor scenario were monitored for a week. RSS measurements were recorded 24/7, in order to encapsulate any environmental factors, such as temperature changes, etc. The results, as shown in Figure 9, indicate that an RSS fluctuation, up to 12 dB may occur (±6 dB from the average RSS value). However, the overall performance of both indoor and outdoor sensors confirm a generally stable operation of ±1–2 dB during 95% of the recorded time. These findings are inline with other scientific work, such as [22,23]. In the aforementioned papers, authors reported an average standard deviation of RSS values ranging from 1 dB to 12.3 dB depending on the device model. The RSS fluctuation range is also affected by human mobility within the study area [24]. These findings partially justify the errors occurring between the measured and simulated RSS values, as analysed previously. Other deviations can be explained due to the unavoidable introduced inaccuracies between the actual and simulated environment. Such inaccuracies may include material constitutive parameters values, object dimensions, geometry and 3D object placements.

### 6.2. Packet Delivery

This section analyses packet delivery, which represents a core performance parameter at the network layer. Table 5, Table 6 and Table 7 depict the simulated packet delivery values as retrieved from Cooja, when the two built-in propagation models of Cooja and the 3D polarimetric ray tracing model of TruNET wireless are utilized. The tables show the expected retrieved number of packets from each node to all others. Obviously, in order to achieve the optimum packet delivery, a strong communication link is required, i.e., a strong RSS value. The 3D ray racing algorithm of TruNET wireless produces a much more realistic estimate of the RSS coverage, indicating significant difference of packet delivery when compared with the other models.

**Figure 10:**
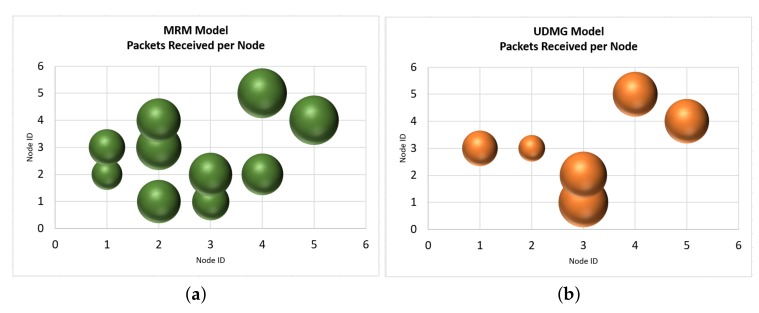
Packets: Cooja built-in models simulation (**a**) MRM Simulation; (**b**) UDMG Simulation.

**Figure 11:**
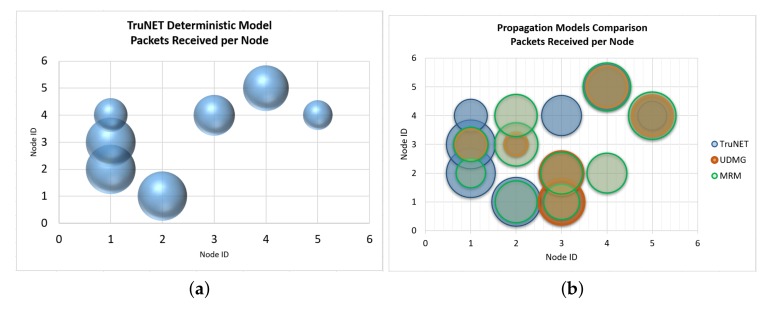
Packets: TruNET vs. Cooja built-in models simulation (**a**) TruNET Simulation; (**b**) Model Comparison Simulation.

Analysis of results’ differences are much more easily spotted in Figure 10 and Figure 11. The most important observations are the following: (a) the packet exchange of Node 3 to Node 4, as well as Node 1 to Node 4, are only predicted by the combined usage of TruNET and Cooja simulators. These communication links are verified by the actual RSS measurements where we recorded RSS values between -79.9 dBm to -81.4 dBm, and they have been recorded. If this RSS value range is compared with results of Figure 2, it is obvious that network packets will be exchanged. (b) Node 3 and Node 2 are not expected to exchange any packets since they fail to establish a communication link (RSS values under the receiver threshold). However, MRM and UDMG models estimate a strong packet exchange. (c) Finally, the combined TrunNET-Cooja OSI cross layer expects that packet delivery between Node 3 and Node 5 will be problematic and packets will be lost, while the other two models expect an almost perfect packet delivery.

Analysis of results indicates the problem that may arise when the wireless network design is based only on the output of a typical network level simulator, where the radio propagation models are not adequate to simulate complex suburban and urban outdoor and indoor scenarios.

Models like MRM and UDMG consider that the transmission range is the range in which the transmitted packet can be received correctly by any node within this range, while the interference range is the range in which the transmission can be heard but the transmitted packet cannot be received correctly. Outside of these two ranges, packets can not be heard. Transmission and reception ratios are random variables that are added to the transmission or reception ratio of a packet to allow the simulation of random errors in the transmission or reception, respectively. Furthermore, a collision will be sensed only in the case that a node attempts to transmit, within the transmission or interference range of a node that is transmitting simultaneously. Then, it depends on the MAC protocol whether the packet will be re-transmitted or dropped. This behaviour is shown from the results of Node 2 and Node 3. While Node 2 can barely communicate with Node 3, the fact that they are within the transmission range of each other (even at the edge) is enough for MRM and UDMG to estimate a strong packet exchange. This behaviour is a significant problem when trying to realistically simulate real deployments. While the nodes seem that they communicate and exchange packets, in real deployment, they cannot communicate at all. This may result in inefficient and insufficient network planning.

On the other hand, if one follows the methodology presented in this work, one can efficiently simulate realistic radio and network conditions and provide accurate and reliable results, valuable for the proper planning of IoT networks.

## 7. Conclusions

This work presented a methodology for addressing the challenges of designing and deploying functional WSN and IoT networks in complex urban environments. Measurements were also performed to verify radio predictions against a real test-bed. It was demonstrated that radio modelling based on 3D polarimetric ray tracing outperforms in accuracy the more simplified radio modelling techniques used by network simulators. When realistic physical layer data are available, network performance prediction in WSNs and IoT networks becomes more reliable.

## Figures and Tables

**Table 1:** Material constitutive parameters of the test environment.

Material	El.Per. (F/m)	L. Tangent
**Concrete**	3.9	0.23
**Wood**	2	0.025
**Brick**	5.5	0.03
**Metal**	1	1,000,000
**Plasterboard**	3	0.067
**Glass**	4.5	0.007

**Table 2:** MRM settings.

Parameter	Value
Receiver sensitivity (dBm)	−100
Max path rays	10
Max refractions	1
Max reflections	3
Max diffractions	0
Refraction coefficient (dB)	−3
Reflection coefficient (dB)	−5
Diffraction coefficient (dB)	−10
Obstacle attenuation (dB/m)	−3

**Table 3:** RSS Comparison—WSN Setup I.

Location	TruNET	MRM	UGDM	Real Measurement
1	−79.20	−97.00	−97.00	−80.00
2	−80.60	−81.77	−97.00	−75.60
3	−70.70	−52.13	−89.00	−78.00
4	−87.90	−97.00	−97.00	−92.00
5	−85.70	−97.00	−89.00	−87.00
7	−78.10	−97.00	−97.00	−87.00
8	−79.00	−97.00	−97.00	−79.50
9	−87.50	−60.77	−97.00	−85.00
10	−85.30	−97.00	−97.00	−98.00
11	−79.90	−97.00	−97.00	−75.50
12	−81.40	−78.96	−97.00	−85.00
13	−78.40	−97.00	−97.00	−85.00
14	−84.60	−52.09	−89.00	−85.00
15	−94.10	−97.00	−97.00	−98.00
16	−74.40	−67.59	−97.00	−86.00

**Table 4:** RSS Comparison—WSN Setup II.

Location	TruNET	MRM	UGDM	Real Measurement
17	−90.00	−82.48	−88.00	−80.00
18	−86.50	−97.00	−97.00	−87.00
19	−63.70	−97.00	−97.00	−66.00
20	−85.00	−97.00	−97.00	−83.50
21	−86.00	−83.78	−88.00	−81.00
22	−93.60	−97.00	−97.00	−95.00
23	−79.20	−52.29	−86.00	−79.00
24	−87.60	−97.00	−97.00	−89.00
25	−76.70	−97.00	−97.00	−86.00
26	−94.00	−97.00	−97.00	−97.00
27	−87.60	−97.00	−97.00	−91.00
28	−57.60	−97.00	−97.00	−67.00
29	−79.50	−52.26	−86.00	−80.00
30	−93.00	−97.00	−97.00	−90.00
31	−95.00	−97.00	−97.00	−93.00
32	−83.00	−97.00	−97.00	−84.50
33	−70.00	−97.00	−97.00	−91.00

**Table 5:** Packet delivery: TruNET wireless.

TruNET	Tx (Packets)	Node 1 Rx	Node 2 Rx	Node 3 Rx	Node 4 Rx	Node 5 Rx	Total Rx
**Node 1**	25,472	-	25,356	25,362	11,752	0	
**Node 2**	25,484	25,360	-	0	0	0	
**Node 3**	25,489	0	0	-	17,378	0	
**Node 4**	25,395	0	0	0	-	21,522	
**Node 5**	25,471	0	0	0	9098	-	
**Total**	127,311	25,360	25,356	25,362	38,228	21,522	135,828
Running Time: 10 min				Tx Rate: 212,185 packets/s			

**Table 6:** Packet delivery: Cooja MRM model.

MRM	Tx (Packets)	Node 1 Rx	Node 2 Rx	Node 3 Rx	Node 4 Rx	Node 5 Rx	Total Rx
**Node 1**	25,471	-	9576	13,030	0	0	
**Node 2**	24,989	18,663	-	20,184	19,117	0	
**Node 3**	25,398	13,821	18,948	-	0	0	
**Node 4**	25,269	0	17,422	0	-	24,428	
**Node 5**	25,343	0	0	0	24,266	-	
**Total:**	126,470	32,484	45,946	33,214	43,383	24,428	179,455
Running Time: 10 min				Tx Rate: 210,783 packets/s			

**Table 7:** Packet delivery: Cooja UDMG model.

UDMG	Tx (Packets)	Node 1 Rx	Node 2 Rx	Node 3 Rx	Node 4 Rx	Node 5 Rx	Total Rx
**Node 1**	25,510	-	0	11,745	0	0	
**Node 2**	25,531	0	-	6540	0	0	
**Node 3**	25,503	23,131	21,280	-	0	0	
**Node 4**	25,486	0	0	0	-	18,994	
**Node 5**	25,467	0	0	0	18,748	-	
**Total**	127,497	23,131	21,280	18,285	18,748	18,994	100,438
Running Time: 10 min				Tx Rate: 212,495 packets/s			

## Abbreviations

The following abbreviations are used in this manuscript:

AP	Access Point
IoT	Internet of Things
MRM	Multi-path Ray-tracer Medium
MDPI	Multidisciplinary Digital Publishing Institute
MS	Mobile Station
OSM	Open Street Map
PRR	Packet Reception Rate
RSS	Received Signal Strength Indication
UDGM	Unit Disk Graph Model
WSN	Wireless Sensor Network
